# Circadian rhythms and post-transcriptional regulation in higher plants

**DOI:** 10.3389/fpls.2015.00437

**Published:** 2015-06-12

**Authors:** Andrés Romanowski, Marcelo J. Yanovsky

**Affiliations:** Laboratorio de Genómica Comparativa del Desarrollo Vegetal, Fundación Instituto Leloir, Instituto de Investigaciones Bioquímicas de Buenos Aires – CONICET (Consejo Nacional de Investigaciones Científicas y Técnicas), Buenos Aires, Argentina

**Keywords:** circadian rhythms, post-transcriptional regulation, Arabidopsis thaliana, alternative splicing, mRNA nuclear export, RNA turnover, polyadenilation, regulation of translation

## Abstract

The circadian clock of plants allows them to cope with daily changes in their environment. This is accomplished by the rhythmic regulation of gene expression, in a process that involves many regulatory steps. One of the key steps involved at the RNA level is post-transcriptional regulation, which ensures a correct control on the different amounts and types of mRNA that will ultimately define the current physiological state of the plant cell. Recent advances in the study of the processes of regulation of pre-mRNA processing, RNA turn-over and surveillance, regulation of translation, function of lncRNAs, biogenesis and function of small RNAs, and the development of bioinformatics tools have helped to vastly expand our understanding of how this regulatory step performs its role. In this work we review the current progress in circadian regulation at the post-transcriptional level research in plants. It is the continuous interaction of all the information flow control post-transcriptional processes that allow a plant to precisely time and predict daily environmental changes.

## Introduction of Cogs and Wheels

We live in a world that spins around its axis with a period of approximately 24 h, which causes daily environmental changes. This has shaped the evolution of organisms living on earth, ultimately leading to the appearance of an endogenous system that helps predict those environmental changes, the circadian clock (Dunlap et al., 2004). The circadian clock mechanism is based on a transcriptional–translational feedback loop (TTFL), consisting of positive and negative elements. This architecture is maintained in all kingdoms of life, although the elements *per se* are different (Brown et al., 2012; Romanowski et al., 2014).

In the case of the workhorse plant model organism, *Arabidopsis thaliana*, the basic layout of the circadian clock operates through the mutual interaction between the MYB transcription factors circadian clock associated 1 (CCA1) and late elongated hypocotyl (LHY), whose levels peak in the morning and repress the expression of the gene encoding PSEUDORESPONSE REGULATOR 1 (PRR1), also known as TIMING OF CAB EXPRESSION 1 (TOC1), whose levels peak in the late afternoon and feedback to repress CCA1 and LHY expression (Nagel and Kay, 2012). In addition, another feedback loop taking place during the morning involves the down-regulation of CCA1 and LHY mRNA levels at midday through the TOC1 homologs known as PRR9 and PRR7 (Farre et al., 2005). During the evening, Lux Arrythmo (LUX, also known as PHYTOCLOCK 1 (PCL1)) recruits early flowering 4 (ELF4) and ELF3 to constitute the evening complex (EC), that acts as a transcriptional repressor targeting PRR9 and LUX itself (Chow et al., 2012; McClung, 2014).

**FIGURE 1:**
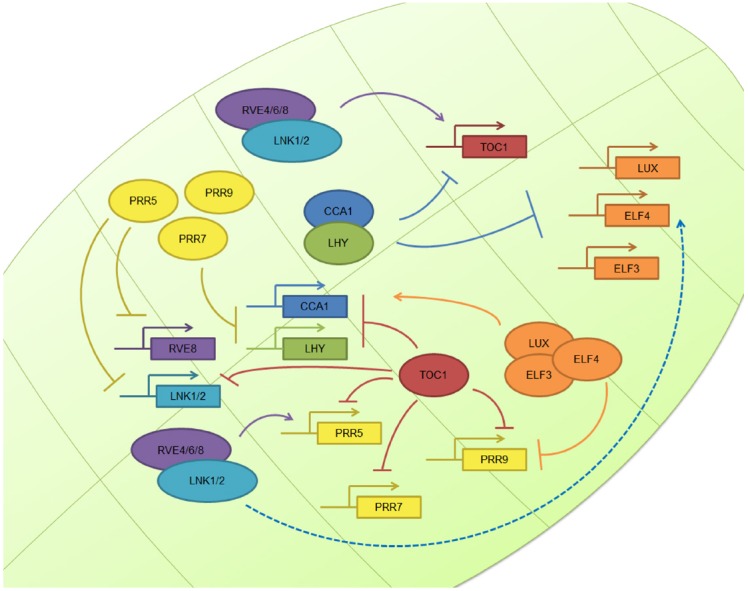
**The plant circadian clock.** The plant molecular clock is based on a TTFL mechanism. The image shows the simplified molecular clockwork mechanism of *Arabidopsis thaliana*: the central loop composed by TOC1, CCA1, and LHY; the morning loop, composed by PRR5, PRR7, and PRR9; the evening complex, composed by ELF3, ELF4, and LUX; and the newly described positive elements, the RVE and LNK family. Other plant clocks are very similar in nature, although there some differences.

While all the above interactions are examples of transcriptional repression, recent evidence indicates that transcriptional activators also play a key role in the regulation of the plant circadian network. In particular, RVE8, RVE6, and RVE4, homologs of CCA1 and LHY, are morning clock factors that activate the expression of afternoon clock genes such as PRR5 and TOC1 (Hsu et al., 2013; Sanchez and Yanovsky, 2013). These two proteins are then degraded by interaction with the F-Box protein Zeitlupe (ZTL; Mas et al., 2003; Fujiwara et al., 2008; McClung, 2014). Recently, four new light regulated clock genes named LNK1–4 have been described. These elements, whose expression peak in the morning, also contribute to activate the expression of afternoon genes such as PRR5 and ELF4 (Rugnone et al., 2013), acting as co-activators in conjunction with RVE8 (Xie et al., 2014). Thus, both transcriptional repression and activation play important roles mediating circadian oscillations in gene expression in *Arabidopsis* (Figure 1). For a complete current review of *Arabidopsis* clock components, see McClung (2014).

The circadian clock is very similar in different plant species, although there are some subtle species-specific differences (Murakami et al., 2007; Higgins et al., 2010; Song et al., 2010; Calixto et al., 2015). Also, even though the main molecular mechanisms of the plant clock have been devised, the complete clockwork is far from complete and more elements will be surely found in the near future. Taken together, this clockwork mechanism orchestrates the oscillation of ∼30% of *Arabidopsis* transcripts (Harmer et al., 2000; Schaffer et al., 2001; Covington et al., 2008; Chow and Kay, 2013). Nevertheless, circadian rhythms are also subject to many other layers of regulation in addition to the transcriptional layer, including epigenetic, post-transcriptional, and post-translational steps. In this review we will focus on post-transcriptional regulation and circadian rhythms and identify areas of research that merit further studies. We apologize to co-workers whose work we could not cite due to article length limitations.

## Post-Transcriptional Regulation

**FIGURE 2:**
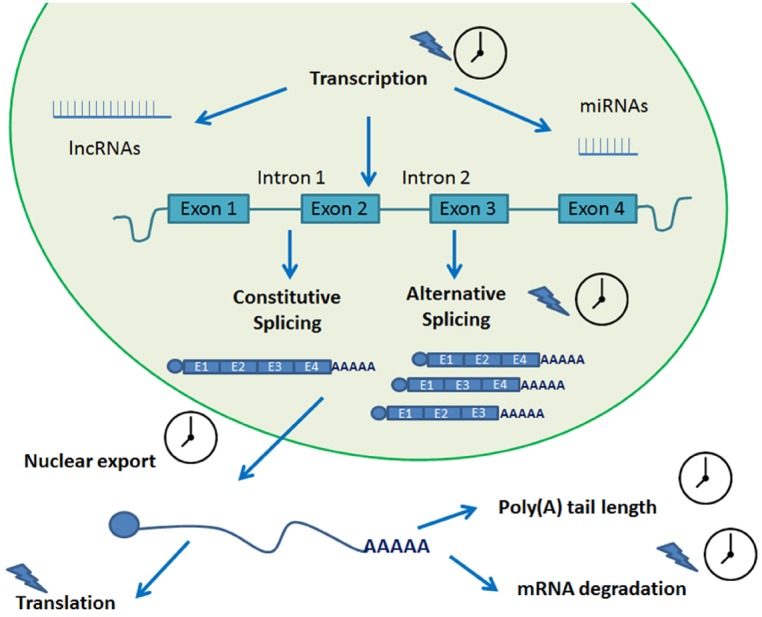
**Post-transcriptional processes involved in circadian biology.** A transcript undergoes several steps of post-transcriptional processing in its journey from transcription to translation. Many of these steps are circadian regulated and help fine tune the circadian rhythms of the plant. A clock indicates that the process is circadian regulated. A blue lightning indicates light regulation.

Several post-transcriptional regulatory steps have been studied in plants. These include pre-mRNA processing, RNA turn-over and surveillance, and regulation of translation (Gallie, 1993; Floris et al., 2009; Kojima et al., 2011). Over the years, evidence has accumulated pointing out the role that each of these events play in circadian gene expression (Kojima et al., 2011; Beckwith and Yanovsky, 2014). These processes all rely on protein–RNA interactions and, not quite unexpectedly, several RNA binding proteins have been shown to cycle in organisms as diverse as algae, insects, and plants (McNeil et al., 1998; Staiger, 2001; Zhao et al., 2004). For a summary of the post-transcriptional mechanisms reviewed in this work, please refer to Figure 2.

### Pre-mRNA Processing

Plant pre-mRNAs exist briefly and are rapidly processed into mature mRNAs. The pre-mRNA molecules are composed of a continuous series of segments, known as exons and introns. Introns must be removed from a precursor mRNA to produce a functional mRNA through a process known as splicing. In addition, exons, which are nucleic acid sequences present in the functional mRNAs after introns have been removed, can be joined in many different ways through alternative splicing (AS; Kornblihtt et al., 2013). Thus, through the process of AS a single gene can actually generate many different proteins (Nilsen and Graveley, 2010).

Alternative splicing seems to be a mechanism to couple changes in environmental temperature and circadian time information (James et al., 2012b; Perez-Santangelo et al., 2013). This type of thermosensitive AS has been observed to occur in *A. thaliana* clock components. One such example is the case of CCA1 during cold acclimation where AS leads to a truncated version of the CCA1 mRNA, CCA1β, that prevents normal binding and function of the active version of CCA1, CCA1α (Seo et al., 2012). Also, in another regulatory strategy, many thermosensitive AS events mediate the inclusion of premature termination codons (PTCs), also referred to as unproductive alternative splicing (UAS), that aim to regulate RNA levels instead of altering protein diversity (Filichkin and Mockler, 2012; Syed et al., 2012). A recent paper shows that environmental stresses alter the accumulation of UAS isoforms and that this reversible channeling allows for a rapid post-transcriptional adjustment of daily oscillations of functional mRNAs (Filichkin et al., 2015).

Pre-mRNA splicing takes place in the spliceosome, a dynamic complex of five small nuclear ribonucleoprotein particles (snRNPs) and additional non-snRNP auxiliary proteins that assemble on the exon-intron boundary sequences known as the donor (5′) and acceptor (3′) splice sites (ss; Wahl et al., 2009). AS results from the alternative use of 5′ ss, 3′ss, exon inclusion or skipping, or intron retention (IR). Splice site recognition and selection plays a key role determining AS patterns and this event is regulated by the interactions between spliceosome components, *trans*-acting proteins, and *cis*-acting sequence signals.

Mutations in spliceosome components that alter normal clock function have been described. SKIP and STIPL1 mutants exhibit a lengthened endogenous period of the biological clock and altered AS rates, including those of clock components (Jones et al., 2012; Wang et al., 2012). Also, a more recent paper has shown that SM-like (LSM) genes, which encode components of the U6 snRNP complex of the spliceosome, are regulated by the circadian clock and control clock function. In this work, it was shown that LSM5 is clock regulated in *A. thaliana* and that several LSM genes cycle in the mouse suprachiasmatic nucleus (SCN). Mutations in both organisms lead to a lengthened period and AS is largely affected in plants (Perez-Santangelo et al., 2014).

Other non-snRNP *trans-*acting splicing factors controlling splice site choice are the serine/arginine rich (SR) and the heterogeneous nuclear RNP (hnRNP) proteins (Matlin et al., 2005). In *Arabidopsis*, the AS of SR and hnRNP proteins is affected by environmental conditions. Also the activity and localization of SR proteins is affected by phosphorylation (Syed et al., 2012). In *Arabidopsis*, some of the best studied hnRNPs are GRP7 and GRP8, components of a slave oscillator coupled to the circadian clock. These two splicing factors auto regulate their own AS and cross-regulate each other to produce UAS isoforms (Schoning et al., 2007, 2008; Syed et al., 2012).

The first evidence of a crosstalk and interplay between circadian rhythms and AS in plants was described a couple of years ago. A mutation in PRMT5, a clock regulated protein arginine methyltransferase, was shown to significantly alter period length by modulating the AS of PRR9 (Sanchez et al., 2010). This constitutes a true feedback loop between AS and circadian rhythms. This protein was also shown to affect circadian rhythms and alter AS rates in the fruit fly, *Drosophila melanogaster* (Hong et al., 2010; Sanchez et al., 2010).

Pre-mRNA also suffers other modifications, such as m^7^G capping. There are no known associations between this process and circadian rhythmicity in plants. However, a recent work has shown that by inhibiting m^7^G-cap methylation and subsequent cap-binding complex association, the endogenous period of the mouse clock is lengthened, thus depicting the importance of RNA-methylation dependent RNA processing for the circadian clock (Fustin et al., 2013). Another type of RNA methylation, m6A methylation, has been recently implicated in circadian biology. When inhibited, this type of methylation which is catalyzed by METTL3, the period of human U2OS cells and mouse SCN slices is slowed. This can be explained by the delay in RNA processing and nuclear mRNA export that was observed upon the knockdown of *Mettl3* (Fustin et al., 2013).

Polyadenylation, a 3′UTR modification, is discussed further below.

### RNA Turn-Over and Surveillance

Stability of transcripts has been long hypothesized to be involved in circadian regulation of gene expression. One such example is the case of CAB1 (LHCB1*3), whose transcript appears to be constant whereas CAB2 (LCHB1*1) and CAB3 (LHCB1*2) show rhythmic transcripts. However, CAB1 promoter driven reporters and nuclear run on assays showed rhythmic transcription, which pointed to the possibility that the steady state of CAB1 mRNA was due to rhythmic regulation of its stability (Millar and Kay, 1991). Also, CATALASE 3 (CAT3) is rhythmically transcribed in constant darkness conditions but transcript levels are kept constantly high at the mRNA level (Zhong et al., 1997). Another interesting case occurs with NITRATE REDUCTASE 2 (NIA2) which is transcribed at a constant rate and is cyclic at the mRNA levels (Pilgrim et al., 1993). In rice, CATALASE A mRNA levels are rhythmic through mRNA stability regulation (Iwamoto et al., 2000). These four examples show that transcripts that are constantly transcribed can be turned into rhythmic mRNA by regulation of its stability and also, that the opposite is also true. All these evidence was however indirect. That changed recently, when the differential mRNA stability of at least two mRNAs was found to be clock controlled in *A. thaliana*: CCR-LIKE (CCL) and SENESCENCE ASSOCIATED GENE 1 (SEN1) transcripts have been found to be circadian regulated at the stability level. The authors have shown that the transcripts are degraded by the downstream (DST) instability determinant pathway and, using *dst* mutants, they have shown a connection between the circadian clock and a sequence-specific mRNA degradation pathway (Lidder et al., 2005). Also, the mRNA stability of the core clock element CCA1 has been found to be light regulated (Yakir et al., 2007). In this work, the authors show that light exposure, particularly red or blue light, causes rapid degradation of *CCA1* mRNA, that is reversed when the plants are transferred to dark conditions. The authors also showed that the *cis* elements involved in this mechanism are located in the coding region and not in the 5′ or 3′ UTR regions.

Another process involved in mRNA turn over that has gained some momentum over the past years is non-sense mediated decay (NMD; Nicholson et al., 2010). As mentioned above, AS sometimes gives rice to PTC containing UAS isoforms and many of these are then degraded by NMD, affecting overall transcript levels and expression (James et al., 2012a; Drechsel et al., 2013; Reddy et al., 2013). In this sense, NMD could sometimes be considered the underlying mechanism for the observed effect of AS. Two of the best characterized examples of gene regulation by this process are GRP7 and GRP8. As we mentioned a few paragraphs above, these proteins bind their own pre-mRNAs inducing AS. The resulting splice variants are NMD-sensitive transcripts that help regulate the transcript levels of both genes (Schoning et al., 2008).

A few years ago there was some evidence that showed that most IR splice variants that had NMD-target features managed to escape NMD turnover (Kalyna et al., 2012). By using *upf1–5* and *upf3-1* single mutants, this work also showed that NMD regulates the transcript levels of plant development genes, transcription factors, RNA processing factors, and stress response genes. This paper also mentions that their evidence points out that PRR9 is regulated by NMD, linking this process to the clock. Last year, a study by Kwon et al., 2014. showed that TOC1 and ELF3 were indeed regulated by NMD, whereas other clock genes were impervious to NMD regulation. It would be interesting to study whether NMD plays a role in fine tuning circadian rhythms. This could be achieved by using the strong NMD defective *upf1–3* mutant. However, this mutation results in lethality due to an adverse autoimmune response. This can be circumvented by using a *upf1–3;pad4* double mutant, which rescues the embryonic lethality of the strong *upf1–3* single mutant, as was recently shown (Riehs-Kearnan et al., 2012).

### mRNA Nuclear Export

The natural flow of information, from DNA to proteins encounters a formidable barrier in plants and all other eukaryotes: the nuclear envelope. The passage of molecules across the nuclear envelope is accomplished through nuclear pore complexes (NPC). This includes the export of RNA molecules complexed with proteins. Most of the RNA transported as RNPs are mRNA and ribosomal RNA (rRNA). The mRNPs can be as large as 100 MDa, which is very large compared to the average size of 60 kDa of regular protein cargoes, and undergo quaternary structure remodeling to pass through the NPC. mRNPs consist of heterogeneous mixes of different proteins packed around a single mRNA molecule (Grunwald et al., 2011; Merkle, 2011; Parry, 2014, 2015). Interestingly, a few years ago, it was found that a protein involved in mRNA export is required for circadian periodicity in *A. thaliana*. Mutants of the E3 ubiquitin ligase HIGH EXPRESSION OF OSMOTICALLY RESPONSIVE GENES1 (HOS1) are affected in cold signaling and exhibit a long period phenotype in a broad range of temperature and light conditions. HOS1 physically interacts with NPC components and is localized in the nuclear envelope. Also, mutant *hos1* plants show elevated accumulation of polyadenylated mRNA inside the nucleus, evidencing a malfunction in nuclear mRNA export. Circadian long period phenotypes were also described for other *hos1*-similar mutants with altered mRNA export: *suppressor of axr1* (*sar1–4*) and *osmotically responsive genes4 (los4-1)*. However, mutants of *hasty mutant (hst-7)*, involved in transport of microRNAs, do not have a lengthened period. This revealed that *hos1* mutants have a period defect resulting from an mRNA export defect (MacGregor et al., 2013). This work shows a true link between circadian rhythmicity and mRNA nuclear export and demonstrated that period lengthening is a general consequence of the disruption of mRNA export in plants.

### Polyadenylation and Regulation of Translation

**FIGURE 3:**
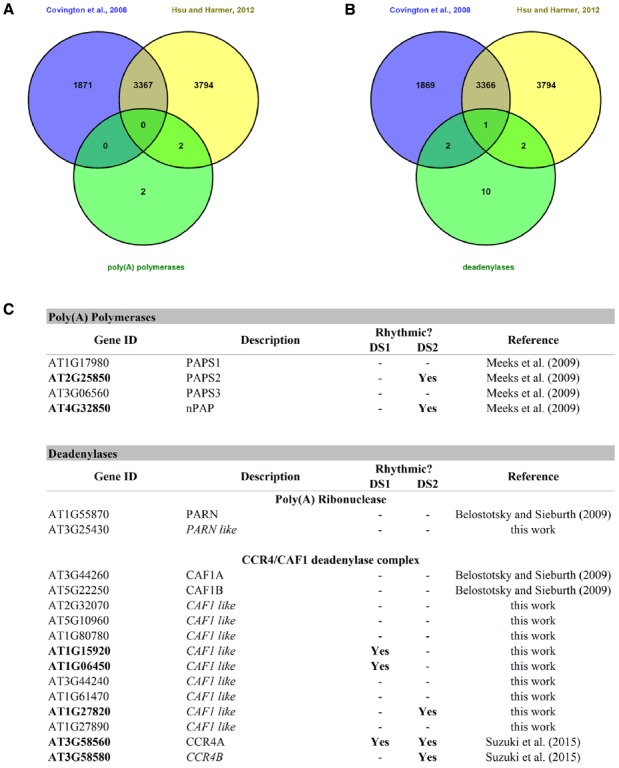
**Rhythmic poly(A) polymerases and deadenylases in *A. thaliana*. (A)** A Venn diagram between the two circadian datasets and the list of poly(A) polymerases. **(B)** A Venn diagram between two circadian datasets and the list of known deadenylases and others found by BlastP homology searches. **(C)** This table shows the rhythmic and arrhythmic poly(A) polymerases and deadenylases of *A. thaliana.* DS1 = Covington + Edwards dataset from Covington et al. (2008); DS2 = circadian dataset from Hsu and Harmer (2012).

Polyadenylation is a key factor controlling mRNA storage, degradation, and translation. As early as 1988, poly(A) tail length has been implicated in circadian regulation in mammals (Robinson et al., 1988). This work showed that the concentration of the neuropeptide vasopressin oscillated in the cerebrospinal fluid of rats and that this correlated with vasopressin mRNA poly(A) tail length, which in turn varied with the time of day. A couple of years ago, it was shown that hundreds of mouse liver mRNAs had poly(A) tail lengths that showed circadian rhythmicity. And although 80% where due to nuclear adenylation coupled to rhythmic transcription, 20% were due to rhythmic cytoplasmic polyadenylation itself. The latter were found to be partly regulated by cytoplasmic polyadenylation element binding proteins (CEBPs; Kojima et al., 2012). Rhythmic changes in poly(A) tail length can result from nuclear and/or cytoplasmic adenylation and deadenylation processes. Several deadenylases and poly(A) polymerases are circadian regulated in mice (Kojima et al., 2012) and could likely contribute to this rhythmic changes in poly(A) tail length. Interestingly, the authors point out that the rhythmicity of poly(A) tail length closely correlates with rhythmic protein expression, and that poly(A) tail length peaks several hours prior to the observed protein expression peak. In *A. thaliana*, four poly(A) polymerases (Addepalli et al., 2004; Meeks et al., 2009) and several deadenylases belonging to the PARN and CCR4/CAF1 complex deadenylase systems (Belostotsky and Sieburth, 2009; Suzuki et al., 2015) have been found. We searched for these genes, and several other homologs that we found by BlastP homology search, in two publicly available circadian datasets (Covington et al., 2008; Hsu and Harmer, 2012) and found that, as was the case in mice, several of them exhibited circadian rhythmicity at the gene expression level (Figure 3). It would be interesting to study poly(A) tail length rhythmicity in plants and also whether the rhythmic plant poly(A) polymerases and deadenylases play a part in this process.

There also seems to be a link between environmental cues and polyadenylation. In 2013 it was reported that two cold induced RNA binding proteins, Cirbp and Rbm3, regulate circadian gene expression by controlling the length of 3′UTR. Under cold conditions, these two proteins bind to proximal polyadenylation sites (PAS) favoring distal PAS selection. The authors also found that proximal/distal PAS selection was, in many cases, under strong circadian regulation (Liu et al., 2013). Whether the same kind of mechanism is or not at work in plants remains to be studied.

Translation is another process that is subject to circadian control. In mammals, it has been shown that AANAT translation is regulated by hnRNP Q by interaction with the 5′UTR of *Aanat*. hnRNP Q is rhythmically expressed and highly correlates with the AANAT phase of expression (Kojima et al., 2011). In *Chlamydomonas reinhardtii*, CHLAMY1, an RNA binding protein with RNA recognition motifs (RRM), regulates the circadian translation of proteins related to nitrogen and carbon metabolism, by recognizing UG repeat sequences in the 3′UTR of its target mRNAs (Waltenberger et al., 2001). This process has not yet been found to be circadian regulated in plants but the studies mentioned are excellent examples to lead the way in plant circadian biology research. It is however, worth to note that a 2003 study found that the translation of a core clock component LHY was light induced. The authors also found that under light:dark cycles, a combination of two simultaneous processes, translational induction and transcriptional repression of LHY expression, could help narrow the peak of LHY protein at dawn (Kim et al., 2003). Later, in 2012, two global studies that showed light regulation of translation were published. Both used sucrose-gradient profiling of ribosomes combined with high-throughput microarray analysis of the ribosome-associated mRNAs to investigate the translational landscape of *A. thaliana* (Missra and von Arnim, 2014). In the first work, by Liu et al. (2012) 4-day-old *Arabidopsis* etiolated seedlings were exposed to white light and harvested at 0 min, 10 min, 0.5 h, 1 h, and 4 h. The translational status of the samples was then examined by polysome profiling analyses. It was found that during photomorphogenesis, genes encoding ribosomal proteins are preferentially regulated at the translational level, and that mRNAs regulated at the translational level have longer half-lives and shorter cDNA lengths. Also a *cis* element, TAGGGTTT, in the 5′ untranslated region was found to correlate with higher translatability (Liu et al., 2012). The second work, by Juntawong and Bailey-Serres, showed that over 1600 mRNAs were differentially translated in response to light availability. In this work, seedlings were grown under long day conditions, shifted to darkness at ZT8, and then re-illuminated. This caused a 17% reduction in polysomes after the dark pulse and an increase in 80S monoribosomes and ribosomal subunits, which was effectively reversed after 10 min of light exposure (Juntawong and Bailey-Serres, 2012). This work identified four distinct groups of genes: dark induced and translated; dark unstable; dark stable and translationally repressed; and non-responsive genes. The first group included mRNAs associated with carbohydrate and amino acid catabolism. The second group was highly enriched in transcription factors and other regulatory proteins. And the third group, which shifted out of polysome complexes and was somehow stabilized, was enriched for plastid targeted proteins and the protein synthesis machinery. This work demonstrated that light:dark transitions have a profound effect on overall and specific translation of mRNAs. There could also be a role for the plant circadian clock in this process. Further studies are needed to answer this question.

### Long Non-Coding RNAs and microRNAs

Non-protein coding RNAs (ncRNAs) are known to be major regulators of cellular processes. In particular, long ncRNAs (lncRNAs), which are generated from the opposite strand of coding or non-coding genes, have a role in transcriptional and post-transcriptional regulation and have been found to be rhythmic in several organisms (Hazen et al., 2009; Coon et al., 2012). In plants, many circadian and light responsive lncRNAs have been found (Hazen et al., 2009; Wang et al., 2014). Some of the lncRNAs have as their natural antisense targets LHY, CCA1, TOC1, PRR3, PRR5, PRR7, and PRR9 (Jouannet and Crespi, 2011). However, the role of these lncRNAs in the central clock remains to be elucidated.

Micro RNAs (miRNAs) have been dubbed the “master regulators of gene expression.” They themselves are subject to a tight regulation at the transcriptional, processing, and localization level (Hamid and Akgul, 2014) and represent another process involved in circadian biology. miRNAs have been shown to be rhythmic in diverse organisms, from plants to mice (Kojima et al., 2011). In plants, it has been shown that the accumulation of several microRNAs (miR171, miR398, miR167, and miR168) has a daily regulation, showing a higher level during the daylight phase of the photoperiodic cycle (Sire et al., 2009). However, this work also showed that this cyclical regulation disappeared upon transfer to constant light conditions indicating that their oscillation was not truly circadian. Another work did succeed in identifying circadian miRNAs in *A. thaliana* using a tiling array that could distinguish only 114 annotated miRNAs (Hazen et al., 2009). Among the several plant circadian miRNAs, this work identified miR157A and miR158A, involved in the regulation of the SQUAMOSA BINDING PROTEIN family regulation; and miR160B and miR167D, involved in the regulation of the AUXIN RESPONSE FACTOR family. These two examples are centered in a rhythmic output of miRNAs. However, a more direct involvement of miRNAs in the circadian clock mechanism has been found in other organisms. For example, disruption of the miRNA biogenesis pathway in *D. melanogaster* severely affects circadian rhythmicity. Using tiling array analyses, several miRNAs involved in normal clock function were identified in the fruit fly head circadian tissue (Kadener et al., 2009). A similar global approach might shed light on miRNA involvement in plant circadian rhythm generation.

### Circadian Bioinformatics Tools Come to the Rescue!

The twenty-first century is the century of big data generation and this is also true in the case of post-transcriptional studies. One can study circadian transcription and pre-mRNA splicing by Nascent-Seq and RNA-Seq (Koike et al., 2012; Menet et al., 2012; Rodriguez et al., 2013), protein–DNA interactions by ChIP-Seq (Huang et al., 2012; Koike et al., 2012; Nakamichi et al., 2012), small RNA biology by tiling arrays and small RNA sequencing (Hazen et al., 2009; Kadener et al., 2009; Yoshitane et al., 2014), and circadian mRNA expression by microarrays and mRNA sequencing (Harmer et al., 2000; Schaffer et al., 2001; Edwards et al., 2006; Covington et al., 2008; Hughes et al., 2012; Koike et al., 2012; Chow and Kay, 2013). Considerations to correctly design RNA-Seq analysis of circadian rhythms have been recently discussed in a very nicely presented methods paper (Li et al., 2015).

Once the data is generated there are several algorithms designed to help identify circadian patterns. Nevertheless, choosing the right algorithm might prove difficult. Some of the most frequently used algorithms have been recently reviewed (Wu et al., 2014). The algorithms reviewed in this work were ARSER (Yang and Su, 2010), COSOPT (Straume, 2004), Fisher’s *G* test (Wichert et al., 2004), HAYSTACK (Mockler et al., 2007), and JTK_CYCLE (Hughes et al., 2010). Using simulated data, some of the most important conclusions were that the sampling rate of the data directly affects the performance of the algorithm and all methods perform equally well when using at least a sampling rate of 2 h/2 days. However, when decreasing the sampling rate to 4 h/2 days or 6 h/2 days, the false discovery rate (FDR) increases. The waveform shape also has implications in the accuracy of detection. For example, while cosine based waveforms are very well detected by the five algorithms, cosine squared, triangle, peak, or squared based waveforms are less accurately identified as sampling rate decreases. Another circadian parameter, phase, is generally detected with an error of approximately 3 h, so this information should be taken cautiously. Fisher’s *G* test does not give phase information. Overall, the authors identify JTK_CYCLE and Fisher’s *G* test as the best algorithms to identify circadian data under different sampling rates. However, each method has its own defined rhythmic signals and they may show preferences for a specific periodic profile, so it is important to determine beforehand the periodic pattern of interest.

Other algorithms were recently compared on the basis that they employed different mathematical methods to identify periodicity (Deckard et al., 2013). The authors compared de Lichtenberg (De Lichtenberg et al., 2005), Lomb–Scargle (Lomb, 1976; Scargle, 1982), JTK CYCLE (Hughes et al., 2010) and persistent homology (Cohen-Steiner et al., 2010). They found that waveform shape had the largest impact on performance, especially under high noise or low sampling rate conditions. In the situations studied in this work JTK_CYCLE was found to be the most versatile algorithm. However, the authors suggest combining the four algorithms together to recover the most comprehensive set of periodic signals in a data set.

Taken together it is advisable to choose the appropriate algorithm based on noise levels, sampling rate and the shape of the periodic pattern of interest. Sampling rate should be the highest possible to obtain the best results. If the pattern of interest is not known, it is advisable to combine various algorithms to obtain a better overview of the circadian data set.

## Final Remarks

We have described several post-transcriptional processes linked with circadian biology. Post-transcriptional regulation adds robustness and complexity to the TTFL based circadian system of plants. However, it is also important not to overlook the fact that circadian rhythms are also subject to epigenetic and post-translational regulation. For example, circadian H3K56ac, H3K9ac, H3K14ac, H3K4me2, and H3K4me3 histone modifications have been described to correlate broadly with circadian mRNA rhythms, indicating a link between chromatin regulation and plant circadian rhythms (Hemmes et al., 2012; Malapeira et al., 2012; Henriques and Mas, 2013). Also, many chromatin remodeling factor seem to be under circadian control of expression (Lee et al., 2015). Post-translational regulatory mechanisms, including protein phosphorylation, stability, and turnover, are also involved in fine tuning circadian rhythmicity and have been recently reviewed (Seo and Mas, 2014). The coordinated phosphorylation of negative elements of the clock, which leads to proteasomal degradation, is essential to determine the pace of the clock. However, a recent work has shown that this final step is not as essential a step as initially thought. Using COP9 signalosome mutants in *Neurospora crassa*, the authors show that in *Δfwd-1* fungi there is no period increase as expected by the lengthened half-life of the negative element FRQ. By using different *frq* alleles they demonstrated that period was determined by the nature of the allele and not the rate of turnover (Larrondo et al., 2015). Another post-translational process implicated in circadian biology is nuclear protein import and, in plants, the import of the circadian clock regulated RNA binding protein AtGRP7 has been studied (Ziemienowicz et al., 2003). This work showed that AtTRN1 is responsible for the nuclear import of AtGRP7 and that the transportin-mediated nuclear input pathway is conserved in man, yeasts, and plants.

There are also other post-transcriptional processes that have been described to be linked to circadian biology in other organisms but not in plants, or that might be under circadian control but have not been yet investigated. An example of the first case is evidenced in small nucleolar RNAs (snoRNAs). This small RNAs guide methylation of both non-coding RNAs and mRNAs. Interestingly, the level of several snoRNA host genes oscillates in the fly brain transcriptome (Hughes et al., 2012), highlighting a possible mechanism by which the clock might couple transcription with this post-transcriptional mechanism.

Bioinformatics tools will become more and more important, and necessary, in global studies of circadian biology as evidenced by the ever increasing complexity of all the circadian layers of regulation. As new techniques to study each layer of post-transcriptional regulation become available, new algorithms must be devised to analyze the data and also, special effort should be put to the development of comprehensive algorithmic tools to integrate the circadian information of each layer of regulation. This would help to better understand circadian regulation with a whole new unifying view, and would prove an invaluable tool not for plant biology alone but for the whole science of chronobiology.

### Conflict of Interest Statement

The authors declare that the research was conducted in the absence of any commercial or financial relationships that could be construed as a potential conflict of interest.
